# Processing speed — A potential candidate cognitive endophenotype for bipolar disorder

**DOI:** 10.1016/j.jadr.2022.100459

**Published:** 2022-12-07

**Authors:** Mirona Letitia Dobri, Taya Prince, Alexandre Paim Diaz, Giovana B. Zunta-Soares, Sudhakar Selvaraj, Rodrigo Machado-Vieira, Thomas D. Meyer, Marsal Sanches, Jair C. Soares

**Affiliations:** UT Center of Excellence on Mood Disorders, Louis A. Faillace, MD, Department of Psychiatry and Behavioral Sciences, The University of Texas Health Science Center at Houston, Houston, Texas, United States

**Keywords:** Bipolar disorder, Endophenotypes, Cognitive dysfunction

## Abstract

**Background:**

Bipolar disorder (BD) is a chronic multifactorial disorder that presents with cognitive impairment as one of its main features, in patients as well as in their first-degree relatives. However, the profile of cognitive dysfunction in BD patients and their relatives is not yet well defined. Various neurocognitive deficits have been proposed as endophenotypes for BD. In the present study, we explored the susceptibility to neurocognitive deficits in BD patients and their siblings compared to healthy controls.

**Method:**

A sample consisting of patients diagnosed with BD (*N*=37), their unaffected siblings (*N*=30) and a healthy control group (*N*=39) was assessed using the Brief Assessment of Cognition for Affective Disorders (BAC-A) battery of tests in various cognitive domains: memory, processing speed, working memory, reasoning and problem solving, and affective processing.

**Results:**

Compared to healthy controls, BD patients and their unaffected siblings showed deficits in attention and motor speed, or processing speed as measured by the Symbol coding task (*p* = 0.008), as well as a similar degree of impairment (*p* = 1.000).

**Limitations:**

The lack of statistically significant findings in the other cognitive domains could be related to differences in task difficulty. Most patients were taking psychotropic medication with varying effects on cognition and being treated as outpatients, implying a currently higher level of functioning, which may limit extrapolation of the sample to the general population of BD patients.

**Conclusions:**

These results support the view of considering processing speed as an endophenotype for bipolar disorder.

## 1. Introduction

Bipolar disorder (BD) is a severe mental illness characterized by a chronically recurring course, with intermittent episodes of depression, mania and hypomania (Ferrari et al., 2016) and varying degrees of neuropsychological deficits and functional impairments (Rowland and Marwaha, 2018), making it one of the leading causes of disability worldwide (Clemente et al., 2015; Kupfer, 2005; Rowland and Marwaha, 2018). With a multifactorial etiology not yet completely understood, BD has seen in the last couple of decades a growing interest in its underlying pathophysiologic mechanisms, genetic and environmental risk factors, and their interactions. This prompted new directions for research (Rowland and Marwaha, 2018) questioning the emphasis on the classic delineation of psychiatric diseases based on overt syndromic behaviors in favor of inextricably exploring them together with their complex underlying genetics (Gottesman and Gould, 2003). As such, endophenotypes have emerged as an important concept for exploring nosological entities along the pathway between genotype and disease.

Endophenotypes are traits found in association with a given disease present in a population, as well as in the first-degree relatives of affected individuals. They are heritable, present in the affected individual regardless of disease activity, and exist in both the affected and non-affected family members at a higher rate than they do in the general population. The disease and the endophenotype also cosegregate within families (Gottesman and Gould, 2003). They could be helpful in the identification of individuals at risk, as well as strategy development and implementation for early intervention. Proposed endophenotypes for BD include neurophysiological, biochemical, endocrinological, neuroanatomical, neuropsychological and cognitive markers (Lenox et al., 2002).

Cognitive impairment is prevalent in BD (Martinez-Aran et al., 2004; Robinson and Nicol Ferrier, 2006), as demonstrated by several studies using various cognitive tests (Bauer et al., 2015; Doyle et al., 2009; El-Badri et al., 2001; Keefe et al., 2014; Martínez-Arán et al., 2004). Patients with BD show cognitive dysfunction compared to healthy controls (HC) both during acute mood episodes (Martínez-Arán et al., 2004; Volkert et al., 2016) and during euthymia (Bora et al., 2009; Cardenas et al., 2016; Torres et al., 2007), but the cognitive domains involved display considerable heterogeneity. When comparing unaffected siblings to HC, studies find the siblings show cognitive impairment in several areas, including verbal memory and sustained attention (Balanzá-Martínez et al., 2008; Bora et al., 2009; Zalla et al., 2004).

## 2. Methods

### 2.1. Participants

The sample consisted of patients diagnosed with bipolar disorder type I (BD-I) or bipolar disorder type II (BD-II), unaffected siblings of the patients and HC. The participants were recruited from the UT Center of Excellence on Mood Disorders outpatient clinic. Permission was requested for contacting the siblings of the participants with BD. HC were recruited through internet or community advertisements and postings. Specific inclusion criteria for patients included: BD probands who met diagnostic criteria for BD-I or II, had a same-gender sibling not affected by BD, were no more than 10 years apart from their siblings in age, and were brought up together with their siblings in the same family. Sibling participants were excluded if they had a diagnosis of BD, major depressive disorder, schizoaffective disorder or schizophrenia, had been diagnosed with alcohol or substance abuse or dependence six months prior to screening, or had taken regular doses of benzodiazepines in the two weeks prior to study participation. Subjects were included in the HC group if neither they nor their first-degree relatives with a previous diagnosis of any DSM-IV Axis I Disorders and if they had never taken psychotropic medication.

The Structured Clinical Interview for DSM-IV Axis I Disorders (SCID-I), a semi-structured interview for the diagnosis of psychiatric disorders following DSM-IV guidelines (First, 2004), was used to interview all participants, establish current mood state and confirm or rule out the diagnosis of BD. Current severity of mood symptoms of the patients was evaluated using the Montgomery Åsberg Depression Rating Scale (MADRS) and the Young Mania Rating Scale (YMRS). The MADRS is a questionnaire for assessing the most common depressive symptoms, consisting of 10 items on a 7-point Likert-scale ranging from 0 to 6. Higher scores indicate a greater severity of depressive symptoms (Montgomery and Åsberg, 1979). The YMRS is one of scales most frequently used to assess manic symptoms. It is an 11-item scale based on the patient’s subjective report and observations made during the clinical interview. Higher scores indicate greater severity of manic symptoms (Young et al., 1978). BD proband euthymia was defined as MADRS < 6 and YMRS < 7.

At the time of screening, 13 patients were depressed, 5 were manic, 1 hypomanic, 2 in a mixed state and 16 were euthymic. The siblings of the patients were euthymic. Thirty-one BD patients were taking psychiatric medication at the time of screening (7 were on lithium, 12 on anticonvulsant mood stabilizers, 14 on antidepressants, 19 on atypical antipsychotics, one on typical antipsychotics, 4 on benzodiazepines, and 3 on stimulants). Of the sibling group, one participant was on stimulant medication. All HC were unmedicated at the time of the study. The study protocol was approved by the UTHealth Institutional Review Board, and informed consent was obtained from all participants.

### 2.2. Cognitive measures

The Brief Assessment of Cognition in Affective Disorders (BAC-A) is a recently published battery of neuropsychological tests developed specifically for patients suffering from affective disorders (Keefe et al., 2014). It is based on and includes the original cognitive tests of the Brief Assessment of Cognition in Schizophrenia (BACS) (Keefe, 2004) and two additional assessments designed to measure the impact of emotional salience on cognition in affective disorders. The short duration and paper-administered approach of the BAC-A supports its suitability as an outcome measure in clinical studies of patients with BD (Bauer et al., 2015; Chen et al., 2019; Keefe et al., 2014; Lee et al., 2018; Rossetti et al., 2019). BAC-A consists of eight subtests assessing verbal memory, working memory, motor speed, verbal fluency, executive functions, attention and motor speed (processing speed), affective processing, and emotional interference (Keefe et al., 2014). One of the subtests, the Emotion Inhibition Test (Emotional Stroop Test) was not administered to the subjects in this study, so it will not be mentioned further.

### 2.3. Statistical analyses

The statistical analysis was performed using IBM SPSS Statistics version 28. For the comparison of the groups with regards to socio-demographic features, we used analyses of variance (ANOVA) and non-parametric tests when appropriate. Analysis of covariance (ANCOVA), with mood state, socio-economic status, and educational level as covariates, was utilized in the comparison of neuropsychological findings across the three groups, with a significance level of 0.05. Post-hoc analyses were performed with Bonferroni adjustment.

## 3. Results

### 3.1. Demographics

The sample consists of 37 patients with BD (7 males, 30 females, mean age = 34.0±9.93 standard deviation [SD]), 30 unaffected siblings (7 males, 23 females, mean age = 35.7±11.80 SD), and 39 HC (11 males, 28 females, mean age = 32.69±10.30 SD). Among the patients with BD, 31 (83.8%) met criteria for BD type I and 6 (16.2%) for BD type II. As shown in Table 1, there were no significant between-group differences in mean age, gender, race distribution or IQ level. Years of education and socioeconomic status showed statistically significant differences between groups (p=0.040 and p=0.009, respectively). Depressed patients had a mean MADRS score of 19.54 (±6.56 SD), whereas patients exhibiting manic, hypomanic or mixed symptoms had a mean YMRS score of 7.0 (±7.59 SD).

**Fig. 1.**
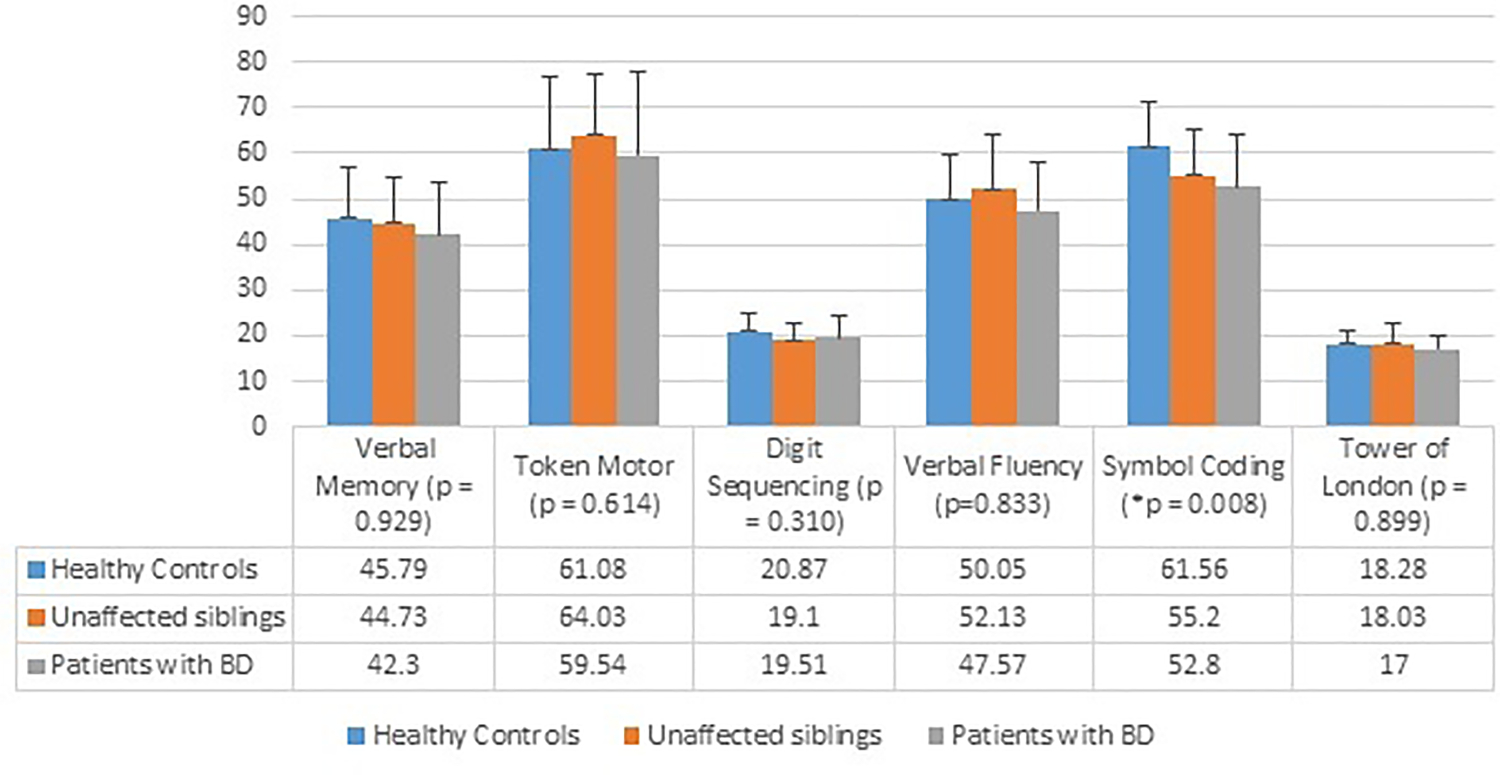
Group differences on the six subtests of Brief Assessment of Cognition. BAC: Brief Assessment of Cognition; BD: bipolar disorder; * Statistical significance with *p* < 0.05.

As shown in Fig. 1, group differences were found in attention and motor speed, measured by the symbol coding task F (2,100) = 5.14, p = 0.008. Adjusted means (±SE) for all tests of the BAC after controlling for mood status, years of education and socioeconomic status are reported in Table 2. Both patients (*p* = 0.015) and siblings (*p* = 0.054) scored lower than HC did. There was no significant difference in the performances between patients and their siblings (*p* = 1.000).

There was no significant difference in performance among the three groups in any of the categories of the Affective Interference Index. The mean raw scores for each group across these categories are reported in Table 3.

## 4. Discussion

This study investigated potential candidate neurocognitive endophenotypes in patients with BD, unaffected siblings, and HC. Our results show decreased performance in the symbol coding task in both the patient and the sibling group compared to the HC group, therefore indicating attention and speed of information processing (Keefe, 2004) as a potential endophenotype for BD. Because siblings also showed some performance deficits as the patients with BD did, our findings suggest that this dysfunction is familial in nature.

This finding is in line with several other studies that have found statistically significant differences in processing speed between patients with BD and their unaffected siblings relative to HC (Antila et al., 2011; Daban et al., 2012; Frantom et al., 2008; Glahn et al., 2010; Thompson et al., 2005). Daban et al. investigated processing speed in patients in BD, unaffected first-degree relatives, and HC in a sample with statistically significant differences in MADRS scores between groups, suggesting impairments that persist during disease remission (Daban et al., 2012). Those results followed earlier reports by Thompson et al., which showed deficits in various cognitive domains, primarily in the psychomotor performance, measured using the Digit Symbol Substitution Test, with over 33% of patients scoring at or below the fifth percentile (Thompson et al., 2005). In a longitudinal study examining the cognitive profile of euthymic patients with BD over a two-year period, deficits in executive functioning and processing speed have been shown to be the most persistent over time (Mur et al., 2008).

More recently, Cardenas et al. conducted a systematic review of studies investigating the neurocognitive performance of euthymic patients with BD and their unaffected relatives. Their results showed deficits in processing speed (all studies found deficits in patients, and 83% of studies found deficits in unaffected relatives), attention (98% in BD and 54% in unaffected relatives), verbal learning/memory (70% in BD, 53% in unaffected relatives), and verbal fluency (81% in BD, 73% in unaffected relatives). These deficits were the most prominent in both categories compared to HC, while unaffected relatives appeared to have preserved working memory, and visual-spatial learning/memory in most studies (Cardenas et al., 2016).

Studies investigating endophenotypes in BD have reported great heterogeneity in the neurocognitive domains being affected. This could be due to studies investigating cognitive impairment in BD displaying considerable methodological variability, as investigators used tests that differ in content, duration, psychometric proprieties, and mode of administration. Furthermore, the definitions and inclusion criteria for the unaffected relatives are heterogeneous, and most studies have compared either patients with BD with HC or unaffected relatives with HC and selected exclusively euthymic patients. Because there is no established instrument of choice for assessing cognitive function in patients with BD, it has been difficult to reach a unified consensus.

Our findings must be interpreted in the context of various methodological limitations. Even though we found significant deficits in processing speed in the patients and their unaffected siblings compared to HC, there was no statistically significant difference in performance in the other cognitive domains measured by the BAC-A, which could be related to differences in task difficulties and the lack of statistically significant difference in the IQ level between groups. Also, the absence of statistically significant results in the other cognitive domains could arguably challenge the positive finding of processing speed deficits. In addition, medication effects could be a contributing factor to the lack of positive findings in most cognitive areas, as the patients were receiving different treatment regimens, and the psychiatric medications used have varying effects on cognition. Atypical antipsychotic agents can positively impact cognitive function (MacQueen and Young, 2003), while mood stabilizers such as lithium have also been demonstrated to not have deleterious effects on cognition (López-Jaramillo et al., 2010) and hypothesized to exert robust neuroprotective properties in animal models (Manji et al., 2000). In addition, the patient sample is treated on an outpatient basis, which implies currently a higher functioning level. Also, because the patient sample is predominantly female, future research should be performed to ensure there is no difference between genders.

## 5. Conclusion

Our study suggests that BD patients and their siblings demonstrate deficits in processing speed when compared to HC. Our results suggest a potential endophenotypic nature of processing speed, with patients with BD performing lower than their unaffected siblings do, who perform lower than do HC. This indicates a possible neurocognitive marker in the high-risk population represented by the siblings of the patients. The findings add to a growing body of evidence pointing to processing speed as a potential endophenotypic trait in BD. However, future research is necessary to demonstrate this and other cognitive deficits as definite endophenotypes for BD.

## Figures and Tables

**Table 1:** Demographic characteristics of subjects by group.

	HC (*n* = 39)	Siblings (*n* = 30)	BD (*n* = 37)	
	*M (SD)*	*M (SD)*	*M (SD)*	*p*

Age	32.69 (10.30)	35.70 (11.86)	34.0 (9.93)	0.510
Education^a^	15.36 (2.32)	14.90 (1.91)	14.03 (2.47)	0.040*
SES^b^	36.13 (16.30)	41.10 (16.75)	28.24 (18.00)	0.009*
WASI				
Vocabulary raw score	52.74 (8.88)	52.03 (12.31)	50.95 (9.64)	0.744
Vocabulary standard score	46.51 (10.07)	45.23 (12.85)	43.49 (10.51)	0.493
Matrix raw score	26.72 (4.07)	25.57 (5.63)	26.38 (3.75)	0.563
Matrix standard score	55.51 (6.37)	54.03 (8.18)	54.59 (7.18)	0.688
FSIQ	102.00 (12.57)	100.07 (15.32)	98.7 (12.31)	0.562
	***n*** (%)	***n*** (%)	***n*** (%)	***P***
Gender				0.637
Female	28 (71.8)	23 (76.7)	30 (81.1)	
Male	11 (28.2)	7 (23.3)	7 (18.9)	
Race				0.731
Caucasian^c^	10 (25.6)	10 (33.3)	11 (29.7)	
Hispanic or Latino	11 (28.2)	9 (30.0)	10 (27.0)	
African American^d^	13 (33.3)	8 (26.7)	11 (29.7)	
Asian	3 (7.7)	1 (3.3)	2 (5.4)	
Multiple^e^	2 (5.1)	1 (3.3)	2 (5.4)	
Unknown^f^	0 (0)	1 (3.3)	1 (2.7)	

X^2^: Chi-Square determined with Kruskal-Wallis Test.

a Years of Education.

b Socioeconomic status, assessed with Hollingshead Socioeconomic Scale, higher values signify a higher SES.

c Non-Hispanic White or Caucasian.

d Black or African American.

e More than one race.

f Unknown or not reported; WASI (Wechsler Abbreviated Test of Intelligence).

* Statistical significance with *p* < 0.05.

**Table 2:** Adjusted and unadjusted means and variability for the six tests included in the Brief Assessment of Cognition.

BAC	HC (N = 39)		Unaffected siblings (N = 30)		BD (N = 37)				p
	Unadjusted M (SD)	Adjusted M (SE)^†^	Unadjusted M (SD)	Adjusted M (SE)^†^	Unadjusted M (SD)	Adjusted M (SE)^†^	F	df	

Verbal memory	45.79 (11.20)	44.30 (1.74)	44.73 (10.26)	43.63 (1.99)	42.38 (10.99)	44.76 (1.97)	0.74	2	0.929
Working memory	20.87 (4.32)	20.55 (0.72)	19.10 (3.83)	18.92 (0.82)	19.51 (4.96)	19.97 (0.81)	1.18	2	0.310
Motor speed	61.08 (15.75)	60.46 (2.73)	64.03 (13.60)	64.04 (3.13)	59.51 (18.49)	60.17 (3.09)	0.49	2	0.614
Verbal fluency	50.05 (9.89)	49.80 (1.71)	52.13 (11.98)	50.73 (1.96)	47.90 (10.40)	48.96 (1.94)	0.18	2	0.833
Speed of processing	61.56 (9.95)	61.27 (1.73)	55.20 (10.01)	55.15 (1.98)	52.79 (11.09)	53.22 (1.96)	5.14	2	*0.008
Executive functions	18.28 (3.060)	17.96 (0.56)	18.03 (4.44)	17.76 (0.64)	16.97 (2.92)	17.55 (0.63)	0.10	2	0.899

HC: healthy controls; BD: subjects with bipolar disorder; M: mean; SD: standard deviation; SE: standard error; BAC: Brief Assessment of Cognition; Verbal memory: number of words recalled in all trials of the List learning task; Working memory: number of correct responses in the Digit Sequencing Task; Motor speed: number of tokens correctly placed in container in the Token Motor Task; Verbal fluency: number of correct responses on the Category instances (animals) and Controlled Oral Word Association tests (F words and S words); Speed of processing: number of correct numerals in the Symbol Coding Task; Executive functions: number of correct responses in the Tower of London test;

† Covariates appearing in the model are evaluated at the following values: Current Mood State = 1.32, Years of education = 14.76, Hollingshead SES_total = 34.78. BAC: Brief Assessment of Cognition;

* Statistical significance with p < 0.05.

**Table 3:** Group differences on BAC-A.

	HC (*N* = 39)		Unaffected siblings (*N* = 30)		BD (*N* = 37)				
BAC-A	*Unadjusted M (SD)*	*Adjusted M (SE)* ^†^	*Unadjusted M (SD)*	*Adjusted M (SE)* ^†^	*Unadjusted M (SD)*	*Adjusted M (SE)* ^†^	*F*	*df*	*p*

Vmtotal	45.79 (11.20)	44.30 (1.74)	44.73 (10.26)	43.63 (1.99)	42.30 (11.27)	44.76 (1.97)	0.74	2	0.929
Toktot	64.95 (14.59)	64.36 (2.65)	67.13 (13.17)	67.01 (3.04)	62.73 (18.24)	63.44 (3.00)	0.36	2	0.698
Tokcor	61.08 (15.75)	60.46 (2.73)	64.03 (13.60)	64.04 (3.13)	59.54 (18.57)	60.17 (3.09)	0.49	2	0.614
tokincor	3.87 (6.64)	3.89 (0.80)	3.10 (2.97)	2.97 (0.92)	3.19 (3.90)	3.26 (0.91)	0.32	2	0.725
tokleft	35.05 (14.59)	35.64 (2.64)	32.97 (13.03)	33.07 (3.03)	37.27 (18.24)	36.56 (3.00)	0.34	2	0.712
dseqtot	20.87 (4.32)	20.55 (0.72)	19.10 (3.83)	18.92 (0.82)	19.51 (5.03)	19.97 (0.81)	1.18	2	0.310
animal	19.59 (4.18)	19.52 (0.78)	21.30 (5.18)	20.76 (0.90)	19.89 (4.89)	20.39 (0.89)	0.61	2	0.541
Fwords	14.85 (4.17)	14.77 (0.72)	15.10 (4.76)	14.64 (0.83)	13.41 (4.67)	13.85 (0.82)	0.32	2	0.723
Swords	15.62 (4.35)	15.50 (0.71)	15.73 (4.92)	15.33 (0.82)	14.27 (3.79)	14.71 (0.81)	0.23	2	0.790
Bacacode	61.56 (9.95)	61.27 (1.73)	55.20 (10.01)	55.15 (1.98)	52.89 (11.38)	53.22 (1.96)	5.14	2	0.008*
London	18.28 (3.06)	17.96 (0.56)	18.03 (4.44)	17.76 (0.64)	17.0 (3.00)	17.55 (0.63)	0.10	2	0.899
Coraff	18.79 (2.01)	18.81 (0.25)	19.2 (0.925)	19.26 (0.29)	18.92 (1.23)	18.84 (0.29)	0.80	2	0.451
Afffalse	0.23 (0.706)	0.21 (0.09)	0.10 (0.305)	0.13 (0.11)	0.30 (0.571)	0.29 (0.10)	0.46	2	0.629
Cornonaff	19.18 (1.74)	19.14 (0.27)	19.2 (1.03)	19.17 (0.31)	18.65 (1.79)	18.71 (0.31)	0.58	2	0.562
Nonafffalse	0.21 (0.469)	0.22 (0.12)	0.20 (0.407)	0.20 (0.14)	0.59 (1.11)	0.56 (0.14)	1.66	2	0.195

HC: healthy controls; BD: subjects with bipolar disorder; M: mean; SD: standard deviation; SE: standard error; BAC-A: Brief Assessment of Cognition for Affective Disorders; vmtotal: number of words recalled in all trials of the List learning task; toktot: total number of tokens placed in container in Token Motor Task; tokcor: number of tokens correctly placed in container in the Token Motor Task; tokincor: number of tokens incorrectly placed in container in the Token Motor Task; tokleft: number of tokens left in container in the Token Motor Task; dseqtot: number of correct responses in the Digit Sequencing Task; animal: number of correct words in the animals category in the Category Instances test; Fwords: number of correct words that begin with the letter F in the Controlled Oral Word Association test; Swords: number of correct words that begin with the letter S in the Controlled Oral Word Association test; Bacacode: number of correct numerals in the Symbol Coding Task; London: number of correct responses in the Tower of London test; Coraff: number of correctly recalled words during the affective learning trials of the Affective Interference test; Afffalse: number of falsely recalled words during the affective learning trials of the Affective Interference test; Cornonaff: number of correctly recalled words during the non-affective learning trials of the Affective Interference test; Nonafffalse: number of falsely recalled words during the affective learning trials of the Affective Interference test;

† Covariates appearing in the model are evaluated at the following values: Current Mood State = 1.32, Years of education = 14.76, Hollingshead SES_total = 34.78.

* Statistical significance with *p* < 0.05.
